# Patterns of Ultra-Processed Food Consumption and Cognitive Performance in Older Adults: A Population-Based Cross-Sectional Analysis from Northern Italy

**DOI:** 10.3390/nu18132074

**Published:** 2026-06-24

**Authors:** Federica Prinelli, Elena Perdixi, Gaia Bonassi, Nithiya Jesuthasan, Sara Bernini, Marco Severgnini, Daniela Martini, Silvia Conti

**Affiliations:** 1 Institute of Biomedical Technologies, National Research Council, Via Fratelli Cervi 93, 20054 Segrate, Italy; nithiya.jesuthasan@cnr.it (N.J.); marco.severgnini@cnr.it (M.S.); or silvia.conti@unife.it (S.C.); 2 IRCCS Humanitas Research Hospital, Via Alessandro Manzoni 56, 20089 Rozzano, Italy; elena.perdixi@humanitas.it; 3 Department of Food, Environmental and Nutritional Science, University of Milan, Via Celoria 2, 20133 Milan, Italy; gaiabonassi@gmail.com (G.B.); daniela.martini@unimi.it (D.M.); 4 Cognitive Psychology Research Section, IRCCS Mondino Foundation, Via Mondino 2, 27100 Pavia, Italy; sara.bernini@mondino.it; 5 Department of Medical Sciences, University of Ferrara, Via Fossato di Mortara, 64/B, 44121 Ferrara, Italy

**Keywords:** NOVA-classification, ultra-processed foods, cognitive function, domain-specific tests, compositional data analysis

## Abstract

**Background**: Given the increasing consumption of ultra-processed foods (UPFs) and the public health importance of cognitive decline in ageing, understanding how UPFs impact cognitive performance is highly relevant. However, evidence in older adults—particularly in Italy—remains scarce, despite the country’s rapidly ageing population, its comparatively low UPF intake, and its distinct Mediterranean dietary context. **Methods**: We analysed cross-sectional data from 809 community-dwelling adults aged ≥ 65 years (59.4% women) participating in the NutBrain population-based cohort. Dietary intake was assessed using a 102-item semi-quantitative food frequency questionnaire, and daily grams of foods were classified according to the NOVA system into groups, which were analysed using a compositional data analysis approach. Global cognition and domain-specific performance were measured using standardised neuropsychological tests. Associations between NOVA groups and cognitive outcomes were estimated using multiple linear regression models adjusted for potential confounders. Gender-stratified analyses were also performed. **Results**: UPFs accounted for 21% of total energy intake, with bakery products as the main contributors. A relative increase of unprocessed or minimally processed foods was associated with better cognitive function (B = 0.36, *p* = 0.014), whereas a greater contribution of UPFs relative to the overall diet was associated with worse global cognitive function (B = −0.26, *p* = 0.003). The strongest associations were observed for episodic memory, particularly among women. **Conclusions**: A higher relative consumption of UPF was associated with worse global and memory-related cognitive performance. Longitudinal and experimental studies are warranted to clarify causality and underlying mechanisms.

## 1. Introduction

In recent decades, global eating habits have undergone substantial transformations driven by economic, social, and cultural factors, as well as by the evolution of the food industry. These shifts have contributed to the growing consumption of ultra-processed foods (UPFs), which can account for up to 56% of total caloric intake in some countries, as in the USA or UK [1,2]. In contrast, intake in Italy remains lower, ranging from approximately 10% [3,4] to 20% [5,6].

The UPF definition was introduced by Monteiro in 2009 [7] through the NOVA classification, which categorises foods according to the extent and purpose of industrial processing. NOVA distinguishes four groups: (i) unprocessed or minimally processed foods, (ii) processed culinary ingredients, (iii) processed foods, and (iv) ultra-processed foods. UPFs are industrial formulations typically containing numerous ingredients that undergo multiple processing steps and often include additives and other non-nutritional components [8]. Importantly, although NOVA categories are defined primarily by the degree of processing, foods within the same group—particularly UPFs—can still differ substantially in their nutritional quality [9].

International epidemiological studies reported that UPF consumption is associated with the increased prevalence of non-communicable diseases [10], including cognitive impairment and dementia [11], although findings remain partially inconsistent. One review [12] reports a significant association with dementia, including Alzheimer’s disease, vascular dementia, and cognitive impairment, while another shows positive associations with multiple sclerosis, Parkinson’s disease, and cognitive impairment, but not with Alzheimer’s disease or dementia [13]. Several biological mechanisms may explain how UPFs could affect the brain. Diets high in refined sugars, unhealthy fats, and additives can promote systemic inflammation, oxidative stress, metabolic dysfunction, and gut dysbiosis, fostering neuroinflammation and impairing neuronal health [14,15].

Given the increasing consumption of UPFs and the public health importance of cognitive decline in ageing, understanding how UPFs impact cognitive performance is highly relevant. However, evidence in Italian older adults remains scarce. Geographic variability in UPF consumption and the heterogeneity in the types of UPFs available across countries further underscore the need for context-specific analyses. Studying UPF consumption in relation to cognition in older Italian adults is particularly important given the lower UPF intake, the rapid ageing of the population, the Mediterranean dietary traditions, and the potentially different types of UPFs consumed compared with other countries.

Furthermore, existing studies have assessed UPF intake in absolute terms, without considering the relative contribution of UPFs to the overall diet—an important methodological limitation given the inherently compositional nature of dietary data, where an increase in one food group necessarily corresponds to a decrease in others, and, thus, should be interpreted as parts of a whole.

Moreover, because cognitive domains follow distinct trajectories of decline and have different clinical implications, distinguishing between global cognitive function and domain-specific performance is crucial. Nevertheless, only a limited number of observational studies have examined associations between UPFs and cognitive domains, with mixed results across memory, executive function, and language [16,17,18].

By addressing these gaps, our study characterises NOVA consumption patterns in an older Italian population. It also evaluates their associations with global cognitive impairment and specific cognitive domains using a compositional data analysis (CoDA) framework.

## 2. Materials and Methods

### 2.1. Study Design, Setting and Participants

This analysis uses cross-sectional data from the Nutrition, Gut Microbiota, and Brain Aging (NutBrain) project [19], a population-based cohort study that focuses on community-dwelling individuals aged 65 and older residing in the Lombardy region of Italy. Data collection occurred between October 2019 and January 2023 through comprehensive assessments, including validated scales and questionnaires to evaluate dietary data, cognitive outcomes, and health-related variables. All baseline evaluations were conducted by trained and certified personnel following standardized protocols. Among the 821 participants enrolled, 809 were included in this analysis; 12 were excluded because they did not complete the entire screening process, specifically dietary assessment (n = 1) or cognitive evaluation (n = 11).

### 2.2. Cognitive Outcomes Data Collection

Cognitive functions were evaluated at baseline using a comprehensive battery of standardized neuropsychological tests designed to assess global cognition and specific cognitive domains, as follows. The global cognitive function was measured by the Mini-Mental State Examination (MMSE) [20], a widely used screening tool with scores ranging from 0 to 30, where higher values indicate a better performance. Functionality in specific cognitive domains was assessed by the following tests: (i) Memory: Free and Cued Selective Reminding Test (FCSRT) [21], Logical Memory Test [22], and the delayed recall of the Rey-Osterrieth Complex Figure Test (ROCF) [23]; (ii) Executive functions: Frontal Assessment Battery (FAB) [24], Phonemic Verbal Fluency [25], Semantic Verbal Fluency [22] and Trail Making Test parts A and B [26]; (iii) Language: Picture Naming Test [27]; and (iv) Visuospatial abilities: ROCF copy condition [23]. All neuropsychological assessment results and clinical records were reviewed by a multidisciplinary panel of neurologists and neuropsychologists, who reached a consensus on any controversial cases. The cognitive test scores were transformed into z-scores by subtracting the average score and dividing the standard deviation of the distribution. Because the MMSE is primarily sensitive to deficits in temporal orientation and memory and less sensitive to impairments in executive and logical functions, to provide a more comprehensive assessment of overall cognitive functioning, a global cognitive composite score was additionally computed by summing domain-specific cognitive test z-scores and re-standardising (z-transformed) the resulting score before its inclusion in the association models.

### 2.3. Dietary Data Collection

Dietary habits over the year before the first visit were evaluated using a 102-item semi-quantitative food frequency questionnaire (SFFQ) [28], administered by a certified dietician. This questionnaire was adapted from the Willett questionnaire, originally used in the Nurses’ Health Study and later modified for the Italian Bollate Eye Study-BEST [29]. The SFFQ covers a wide variety of food categories, including bread, cereals, tubers, eggs, meat, processed meat, legumes, fish, dairy products, cheeses, vegetables, fruits, sweets, oils, condiments, sauces, beverages, coffee, alcohol, sugars, and miscellaneous items. The participants were asked to report how often they consumed a standard portion of each food item on a seven-point scale ranging from ‘never to rarely’ (less than once per month) to ‘4 to 5 times per day.’ The intermediate options included consumption frequencies of 1 to 3 times per month, 1 to 2 times per week, 3 to 4 times per week, once per day, and 2 to 3 times per day. Daily intake (g/day) of each food was calculated by multiplying the reported consumption frequency by the corresponding portion size.

### 2.4. NOVA Classification

The food items in this study were grouped according to the NOVA classification system, which includes four categories: unprocessed or minimally processed foods (NOVA1), processed culinary ingredients (NOVA2), processed foods (NOVA3), and ultra-processed foods (NOVA4). The assignment of food items to the four NOVA categories was carried out by one researcher (G.B.) and subsequently checked by two additional researchers (S.C. and F.P.) after an extensive review of the literature and available classification guidelines. Detailed information about the food items in each NOVA category is provided in Supplemental Table S1. To evaluate the total consumption of each NOVA group in grams per day, we summed the weights of the food items included in the group. Then, the weight ratio was used to estimate the daily proportion of each NOVA group relative to the total weight of the diet (in grams) and was used in the analyses. This approach accounts for food items that do not provide energy (e.g., artificially sweetened beverages) and non-nutritional factors related to food processing (e.g., newly formed contaminants, food additives, and alterations to the structure of raw foods) [30]. Additionally, for descriptive purposes, the daily energy intake for each NOVA group was obtained by summing the calorie content of all foods within each category and then expressing this value as a percentage of total daily energy intake. This relative measure provides information on the contribution of ultra-processed foods to each participant’s overall diet, while also accounting for individual differences in caloric intake and allowing comparison with other studies.

### 2.5. Application of CoDA to NOVA-Classification

To consider the compositional nature of dietary data—where components represent parts of a whole and thus convey relative rather than absolute information—NOVA groups were analysed using a CoDA approach based on log-ratio transformations [31]. This methodology has already been applied in nutritional epidemiology [32,33,34]. We aimed to evaluate the relative dominance of each NOVA group with respect to the others. We used additive log-ratios (alr) (using daily grams of NOVA groups), which are mathematically equivalent to simplified *pivot balances* [35,36]. Given that NOVA2 represented a very small proportion of total intake, we combined processed culinary ingredients (NOVA2) with processed foods (NOVA3) into a single category (NOVA2–3) prior to transformation. The resulting three-part diet composition therefore consisted of NOVA1, NOVA2–3, and NOVA4. For a D-part composition ($x_{1}$, $x_{1}$, …, $x_{D}$), the alr is defined as:

$$alr\left(x\right)\left[ln\left(\frac{x_{1}}{x_{D}}\right)ln\left(\frac{x_{2}}{x_{D}}\right),\ldots ,ln\left(\frac{x_{D-1}}{x_{D}}\right)\right]$$

In our primary analysis, we calculated two alr coordinates [33,37] using NOVA4 as the denominator, and these were included as predictors in the regression models.

Let y represent the outcome. The model can be written as:

$$f\left(y\right)=B_{0}+B_{1}\cdot ln\left(\frac{NOVA1}{NOVA4}\right)+B_{2}\cdot ln\left(\frac{NOVA2-3}{NOVA4}\right)+\sum_{i}B_{i}\cdot z_{i}$$

where $z_{i}$ represents covariates.

In this framework, for example, the coefficient B_1_ represents the expected change in the outcome when NOVA1 increases and NOVA4 decreases, while keeping all the other terms in the model constant. Due to the compositional constraint, NOVA2–3 will decrease as well by the same factor as NOVA4. To further estimate the relative dominance of NOVA4, we computed two other alr using NOVA1 as the denominator and fitted additional regression models [34].

### 2.6. Non-Dietary Data

For this analysis, we examined the following covariates: gender (men and women), age (continuous); education (categorised as university, high school, middle school and primary school or below); the number of daily medications used (as a proxy for comorbidities); and smoking habits (classified as never, former or current smokers). Depressive symptoms were evaluated using the 20-item Centre for Epidemiological Studies Depression Scale (CES-D) [38], which focuses on participants’ feelings and behaviours during the week before the interview. Waist circumference was measured to the nearest 0.5 cm using a flexible measuring tape (SECA 201) at the midpoint between the 12th rib and the iliac crest. Physical activity was assessed via the International Physical Activity Questionnaire (IPAQ) [39]. Participants were categorised according to international guidelines [40] as inactive (no physical activity; score = 0), minimally active (performing one low-intensity activity such as slow walking; score = 1), moderately active (performing more than one light or moderate activity such as slow swimming, dancing or cycling; score = 2) or vigorously active (performing high-intensity activities such as hiking, skiing, fast swimming or running; score = 3). Daily energy intake was estimated using the Italian Food Composition Databases for Epidemiological Studies in Italy [41], combined with MetaDieta software (Meteda s.r.l., San Benedetto del Tronto, Italy). Following previous literature [42], we operationalised the construct of social isolation as a composite measure of seven types of social connectedness, using data derived from ad hoc questions and the Cognitive Reserve Index Questionnaire (CRIq) [43]. Indicators included household composition (0 = more than one co-habitant, including the respondent; 1 = living alone); membership of voluntary organizations (0 = yes; 1 = no), involvement in church or other community groups (0 = yes; 1 = no), provision of care for nephews or elderly relatives (0 = yes; 1 = no), and participation in social and cultural activities, including going to the cinema, travelling, and visiting museums or exhibitions (each coded as 0 = yes; 1 = no). A total social isolation score ranging from 0 to 7 was calculated by summing the individual items, with higher scores indicating greater isolation. For analytical purposes, the composite score was categorized into three levels of social connectedness: mostly integrated (scores 0–2), poorly integrated (scores 3–4), and mostly isolated (scores 5–7).

### 2.7. Statistical Analysis

Descriptive statistics were used to characterize the study sample, with the means and SD reported for continuous variables and the frequencies and percentages for categorical variables. Differences in participant characteristics between men and women were assessed using t-tests for continuous variables and chi-squared tests for categorical variables. To estimate the associations between dietary composition and cognitive performance, we fitted linear regression models using the MMSE score (considered as a continuous variable), domain-specific tests, and global cognitive z-scores as outcomes. The NOVA components were entered into the models using the alr transformations described above. Regression coefficients (B) and standard errors (SEs) were reported, with statistical significance indicated by asterisks. All models were adjusted for age, gender, educational level, smoking status, physical activity, medication use, total energy intake, depressive symptoms, waist circumference, and social isolation. To assess potential effect modification by gender, interaction terms between alr and gender were tested, followed by gender-stratified analyses. To minimise the possibility that severe cognitive impairment may affect dietary choices or reporting accuracy, we performed a sensitivity analysis excluding participants with a corrected MMSE score below 22. This is the cut-off point recommended by the Magni criteria [20] for adults aged 65 years and older, indicating a cognitive deficit. All analyses were conducted using Stata 19 (StataCorp, College Station, TX, USA).

## 3. Results

### 3.1. Participant Characteristics

The cohort included 809 individuals aged 65–94 years (mean age 73.3 ± 6.2 years), of whom 59.4% were women. The cohort characteristics are presented in Table 1. Compared with men, women were less educated, less physically active, more often never smokers, used fewer medications, reported more depressive symptoms, and had a lower waist circumference.

### 3.2. Patterns of NOVA Food Consumption

**Figure 1:**
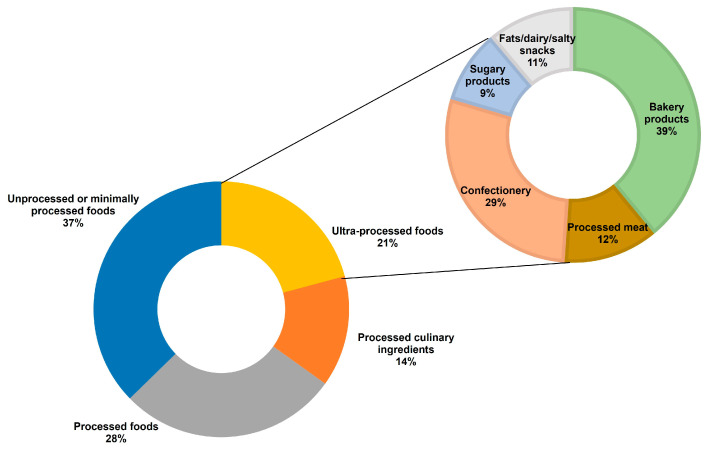
Contribution of NOVA food groups (in %) to total energy intake in the study population and main contributing food sources to the total energy intake for ultra-processed foods (n = 809).

With respect to dietary habits, Table 1 and Figure 1 show the contribution of each NOVA group to total energy intake (%). In the overall sample, unprocessed or minimally processed foods (NOVA1) were the most consumed, contributing to 37% of the daily energy intake, followed by processed foods (28%). UPFs (NOVA4) accounted for 21% of the mean energy intake. Within UPFs, bakery products and confectioneries were the most frequently consumed subgroups, contributing 39% and 29% of UPF intake, respectively.

Women reported a significantly greater proportion of calories from NOVA1 and NOVA2, whereas men derived a greater share of their diet from NOVA3 (*p* < 0.001). The contribution of UPFs accounted for roughly one-fifth of total energy intake in both men and women. Ultra-processed food consumption patterns were broadly similar between men and women (a trend for processed meat, which is consumed more by men *p* = 0.06), with both deriving a substantial proportion of their energy from bakery products and confectionery.

### 3.3. Associations with Global Cognitive Performance

**Figure 2:**
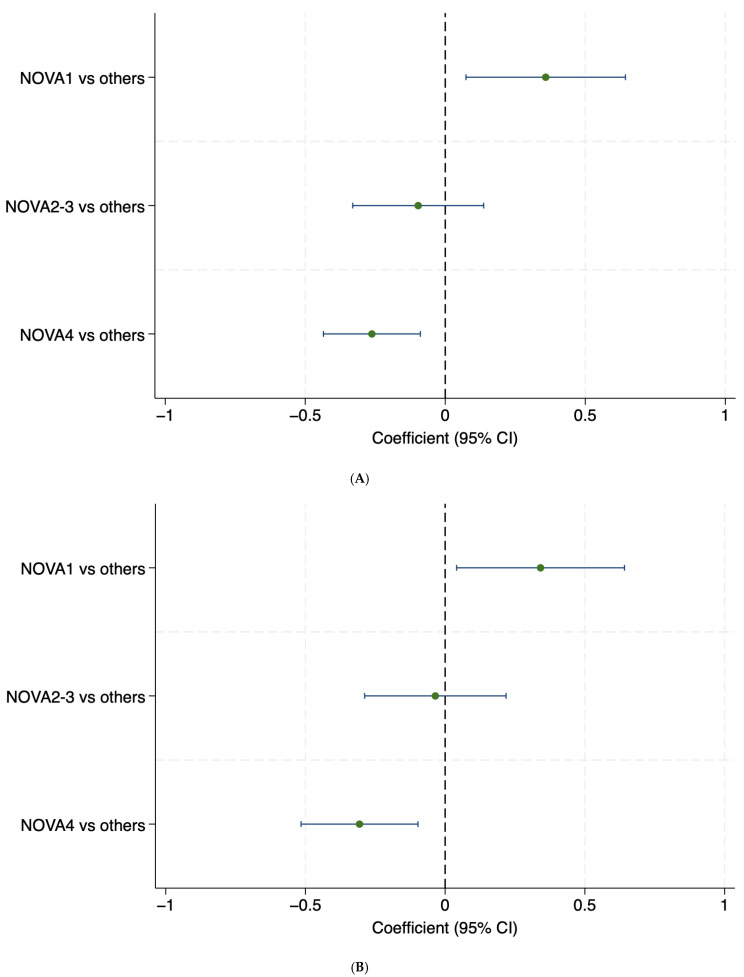

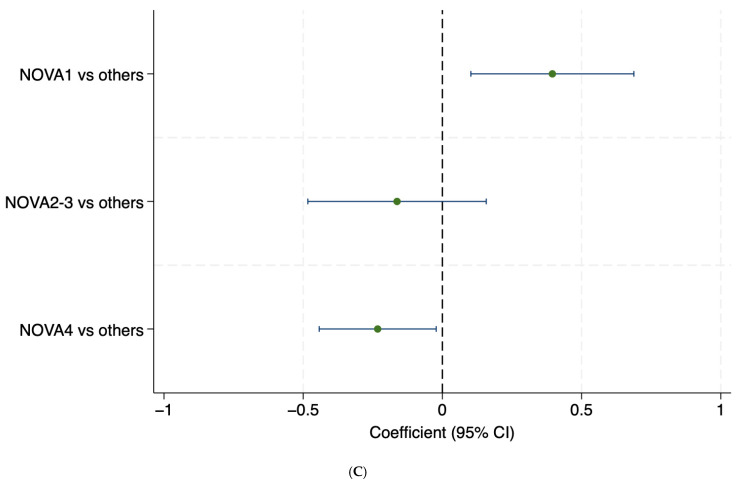
Forest plot of the linear regression coefficients (B) and 95% Confidence Intervals (CIs) for associations between NOVA group (alr) and global cognitive function measured by MMSE (continuous score). Legend: CI, confidence interval. The models also include terms for age, gender, education, practising sports, smoking, depressive symptoms, daily number of drugs, total energy intake, waist circumference, and social isolation. (**A**) all participants, (**B**) men, (**C**) women.

Figure 2A–C and Supplementary Table S2 present the associations between NOVA food group balances expressed as alr and MMSE scores. Each coefficient reflects the association between a specific NOVA balance and cognitive performance while holding constant the proportional relationships among the remaining NOVA components. In the multiple linear regression model, a higher dominance of NOVA1 relative to other groups was positively associated with MMSE score (B = 0.36, *p* = 0.014). We did not find an association with NOVA2–3; conversely, an increase of NOVA4 (B = −0.26, *p* = 0.003) was negatively associated with MMSE scores (Figure 2A). We did not find a statistically significant interaction between gender and NOVA groups; however, gender-stratified analyses revealed that a positive association between NOVA1 and MMSE score was present in both men and women (B = 0.34, *p* = 0.026 in men; B = 0.40, *p* = 0.008 in women). An increase of NOVA4 was negatively associated with MMSE in both men and women (B = −0.31, *p* = 0.004 in men; B = −0.23, *p* = 0.030 in women) (Figure 2B,C).

### 3.4. Associations with Cognitive Domains

Across cognitive domains (Table 2), Increasing NOVA1 relative to other groups was positively associated (*p* < 0.05) with all FCSTR indices (with a borderline association for delayed total recall, *p* < 0.10), with Picture Naming Test (B = 0.178), and with the global cognitive composite score (B = 0.158). In contrast, a greater relative contribution of NOVA4 was negatively associated with FCSRT immediate total recall (B = −0.120) and delayed free recall (B = −0.110). Borderline associations were also observed for FCSRT delayed total recall (B = −0.086) and for the global cognitive score (B = −0.079). A borderline negative association was additionally found between NOVA2–3 and the Picture Naming Test (B = −0.114), ROCF copy (−0.137), and the global cognitive score (B = −0.079).

Gender-stratified analyses revealed some differences. In women, relative dominance of NOVA1 and NOVA4 was significantly associated with nearly all FCSRT tests, whereas in men the association with NOVA1 was significant only for FCSRT immediate recall (B = 0.191). Women also showed a significant association between NOVA4 and Logical Memory test (B = −0.099), which was not observed in men. A significant association (*p* < 0.05) between NOVA1 and Picture Naming Test scores emerged in both men (B = 0.212) and women (B = 0.157), while the association with NOVA2–3 was significant in men only (B = −0.124). Finally, NOVA4 was negatively associated with the global cognitive composite score in women only (B = −0.117).

### 3.5. Sensitivity Analysis

To minimise the possibility of reverse causation we carried out a sensitivity analysis excluding participants with severe cognitive deficit (MMSE < 22, n = 12). The associations between NOVA balances and global cognitive function remained largely consistent with those observed in the main analysis. A higher dominance of NOVA1 remained positively associated with global cognitive performance, though borderline significant (B = 0.22, *p* = 0.067) in the entire sample. A higher NOVA4 dominance was associated with poorer cognitive performance (B = −0.16, *p* = 0.031). In gender-stratified analyses, these trends persisted, but those with the relative NOVA4 dominance lost significance in women (Supplementary Table S3).

## 4. Discussion

### 4.1. Patterns of Consumption of Ultra-Processed Foods

In this cohort, UPFs accounted for 21% of total energy intake, a proportion lower than that reported in countries such as the United States [44], the United Kingdom [45] or the Netherlands [46], where UPFs often contributed more than 50% of total energy intake. However, our findings are broadly consistent with previous Italian studies reporting estimates between approximately 10% [3,4] and 20% [5,6], although data specifically referring to older people remain limited. Ruggiero et al. [6] found that UPFs accounted for 14% of total energy intake in participants aged over 65 years in the online UFO survey. A plausible explanation for the higher proportion observed in our cohort is that web-based surveys may underrepresent older adults, particularly those with limited digital access or literacy [47], thereby yielding a sample that is not fully representative of the broader older population (only 9% of their cohort was over 64 years of age). Moreover, our participants were all residents of the Milan area, whereas Ruggiero et al. included individuals from across Italy; differences in geographical setting, dietary patterns, and socio-demographic composition may therefore have contributed to the observed discrepancies. In our study, UPFs were mainly represented by bakery products such as crackers, plain biscuits, and breadsticks and confectionery items, which included brioches, pastries, cakes, chocolate, and packaged crisps. These findings closely mirror those of the UFO study [6], in which packaged biscuits, chocolate, crackers, *taralli*, breadsticks, *frisella,* rusks, and ready-to-heat pizza and focaccia were identified as the primary contributors to the total energy from UPFs. Overall, these findings suggest a context-specific pattern of consumption of UPFs in Italy.

### 4.2. Association Between NOVA Food Categories and Cognitive Outcomes

We found that greater intake of unprocessed or minimally processed foods (NOVA1) relative to the other groups was associated with higher MMSE scores, whereas the reverse pattern (UPFs) was associated with lower scores. Our results are supported by evidence on UPFs and cognitive impairment and dementia. For example, a prospective cohort study using data from the UK Biobank found that UPF consumption was associated with an increased risk of all-cause and vascular dementia [48]. Another study among individuals younger than 68 years reported that higher UPF consumption was associated with an increased risk of Alzheimer’s disease [49]. Furthermore, a prospective meta-analysis of observational studies found that higher UPF intake was associated with a 44% increased risk of incident dementia [12].

Considering the cognitive domains, our findings suggest that NOVA groups were selectively associated with episodic memory, with women appearing particularly sensitive to these associations. In contrast, executive functions, language, and visuospatial abilities showed limited or inconsistent associations. To our knowledge, very few studies have examined the role of NOVA groups across cognitive domains. The Brazilian Longitudinal Study of Adult Health [18] showed that high UPF consumption was associated with a faster decline in executive functioning. Two additional studies in older U.S. adults, the Health and Retirement Study and the NANHES, suggested potential associations between high UPF intake and poorer executive function, as well as worse language and executive functioning [16,17]. Conversely, a study among older Dutch adults explored associations between UPFs and global cognition and domain-specific tests but found no significant association [50]. Also, Li and colleagues [48] did not find differences in prospective memory, reasoning score, and reaction time by UPF consumption level.

### 4.3. Biological Interpretations

Although these findings should be interpreted with caution, they are consistent with plausible neurobiological mechanisms. UPFs are industrial formulations that combine extracts of original foods with industrial ingredients and often contain high levels of fat, salt, and added sugars, as well as additives such as emulsifiers, sweeteners, colours, and preservatives [8], which have been suggested to promote pro-oxidative and pro-inflammatory effects and potentially affect neuronal function [14]. UPF consumption has also been associated with alterations in gut microbiota composition and intestinal integrity, potentially contributing to dysbiosis [15,51], which may play a role in neuroinflammatory and neurodegenerative processes [52]. In this context, the pattern observed in the FCSRT supports the hypothesis that episodic memory processes—the ability to recall past events and experiences [53] could be particularly susceptible to these mechanisms. This interpretation is consistent with evidence linking Western dietary patterns, typically rich in fats and added sugars, to impaired hippocampus-dependent learning and memory [54,55]. Animal studies provide further mechanistic insights: rodents fed high-fat diets show reduced synaptic plasticity and impaired memory function, alongside alterations in inflammatory markers in the hippocampus and amygdala [56,57] affecting hippocampal function [58]. Furthermore, the accumulation of dietary advanced glycation end-products (AGEs), glycotoxic compounds generated during high-temperature industrial processing and present in several UPFs including bakery products [59], has been associated with faster cognitive decline, particularly in episodic memory, in epidemiological studies [60,61].

Given the heterogeneity of UPFs, the observed associations may be partly driven by the predominant food groups in this cohort, namely bakery products and confectionery; however, this remains speculative and was beyond the scope of the present analysis.

### 4.4. Other Possible Explanations

Because the cross-sectional design does not allow us to determine whether cognitive impairment leads to higher UPF intake or vice versa, reverse causation may partly explain our findings. A bidirectional relationship between UPFs and cognitive function is also plausible. Eating difficulties, changes in appetite, and shifts in food preferences are common in individuals with cognitive impairment and dementia [62]. People with dementia may overeat, crave sweet foods and soft or sweet drinks, or rely on a limited number of foods. Therefore, individuals with early cognitive impairment and memory complaints may be more likely to prefer ready-to-eat products and highly palatable foods, such as UPFs. Alternatively, family members or caregivers may choose quick, packaged options to simplify daily routines. These changes may occur years before the onset of clinical dementia [63] in individuals with an initial cognitive deficit. While future longitudinal studies that exclude prevalent dementia cases are needed to minimise bias and clarify causality, the consistency of these associations after excluding participants with moderate-to-severe cognitive deficits (MMSE > 22) suggests that reverse causation is unlikely to fully account for the observed relationships. Another possible explanation is that individuals who regularly consume UPFs may have comorbidities or engage in less healthy behaviours, such as lower physical activity or reduced social participation, which are themselves associated with poorer cognitive outcomes. However, adjusting our models for these potential confounders helped isolate the independent association between UPF intake and cognitive performance, suggesting that our observations are robust against this source of confounding.

### 4.5. Gender-Stratified Analyses

Our gender-specific analyses showed that, compared with men, women derived a higher proportion of energy from unprocessed or minimally processed foods. This finding is consistent with previous evidence indicating that women are more likely to adopt healthier dietary behaviours, such as limiting high-fat and high-salt foods and choosing fibre-rich options such as fruit, vegetables, and whole-grain products. In contrast, men more commonly consume red and processed meat, eggs, alcohol, and foods high in added sugars, as well as greater quantities of ready-to-eat processed products [64,65,66]. Associations between NOVA groups and cognitive outcomes were more pronounced in women across nearly all memory measures, suggesting that they may be more vulnerable to UPFs than men. Similarly, findings from the US Health and Retirement Study reported stronger associations between UPF intake and language impairment among women [17]. Potential explanations include sex differences in neuroanatomy [67], sexual hormones, and metabolic [68] and gut microbiota responses to the same diet [69], although these mechanisms remain to be clarified. Nevertheless, although we adjusted for several relevant confounders, we cannot exclude the possibility that differences in individual characteristics, such as physical activity, depressive symptoms, health status, or other lifestyle factors, may partly account for the observed-specific patterns; therefore, results should be interpreted with caution.

### 4.6. Strengths and Limitations

This study presents some limitations. In addition to the cross-sectional design and potential reverse causation, which have already been discussed above, all the questionnaires, including the SFFQ, relied on self-reporting and are, therefore, subject to recall bias. A further limitation is that the dietary assessment tool was not designed or validated to identify ultra-processed foods according to the NOVA classification. Thus, many UPF items were not included (e.g., pre-cooked meals, dietetic products); this may have resulted in non-differential misclassification of UPF intake, which could have biased the estimate towards the null, weakening the observed associations.

Furthermore, because no universal consensus exists for the classification of certain food items, misclassification cannot be excluded. For potentially ambiguous items, particularly those that could fall into NOVA4, we adopted a conservative approach by assigning them to NOVA3, thereby reducing the risk of overestimating UPF exposure.

In addition, despite adjusting for a wide array of relevant potential confounders, residual confounding due to measured and unmeasured factors cannot be entirely ruled out. Finally, the NOVA classification is widely used due to its practicality and its ability to facilitate comparisons across studies; however, it remains a subject of ongoing debate, as it is based on multiple criteria that are not always objectively quantifiable, including the degree of processing, formulation characteristics, and the presence of cosmetic additives. Accordingly, the present findings should be interpreted with caution. Given the substantial heterogeneity within the NOVA4 category, future analyses should aim to explore associations by considering more specific sub-categories of UPFs, to better identify the components potentially driving the observed associations. In addition, further work is warranted to develop more refined classification frameworks that account for both processing levels and nutritional quality [70,71].

The study also presents several strengths. The first one lies in the application of compositional data analysis (CoDA) to evaluate NOVA food categories in relation to cognitive outcomes, for the first time. Because dietary data represent parts of a whole, traditional analytical methods may yield misleading results by failing to account for the inherent interdependence among food groups. CoDA addresses this limitation by treating the proportions of unprocessed foods, culinary ingredients, processed foods, and ultra-processed foods as components of a single composition. This approach minimises multicollinearity, emphasises ratios between food categories, and offers a more integrated understanding of how changes in one NOVA component correspond to shifts in the others and, ultimately, to cognitive performance.

Additional strengths include the extensive baseline assessment, which enabled the collection of a broad set of covariates, and the use of a well-characterised, community-dwelling, population-based cohort, enhancing the generalisability of the findings to free-living older adults. Cognitive function was assessed with standardised neuropsychological instruments, and all questionnaires were administered by trained staff, thereby ensuring high data quality and supporting accurate recall of dietary and behavioural information.

## 5. Conclusions

In conclusion, in this cohort of older Italian adults, UPFs accounted for 21% of total energy intake, a proportion lower than that reported in many other countries, likely reflecting the persistence of Mediterranean dietary traditions. A diet characterised by a higher contribution of UPFs was associated with worse cognitive performance, particularly in episodic memory. Future longitudinal and experimental studies are needed to clarify the temporal sequence of these associations and the possibility of reverse causation. Even in the absence of definitive causal evidence, clinical and public health strategies that promote diets rich in minimally processed foods while limiting UPFs consumption may represent a practical approach to support cognitive function in older adults.

## Figures and Tables

**Table 1:** Participants’ characteristics by gender (n = 809).

	All (n = 809)	Men (n = 328, 40.57%)	Women (n = 481, 59.43%)	*p*-Value
Sociodemographic characteristics				
Age				0.093
65–69	290 (35.85)	101 (30.79)	189 (39.29)	
70–74	196 (24.23)	84 (25.61)	112 (23.28)	
75–79	172 (21.26)	78 (23.78)	94 (19.54)	
80+	151 (18.67)	65 (19.82)	86 (17.88)	
Educational level				<0.001
Primary school or less	108 (13.35)	32 (9.76)	76 (15.80)	
Middle school graduate	211 (26.08)	66 (20.12)	145 (30.15)	
High school	340 (42.03)	155 (47.26)	185 (38.46)	
University	150 (18.54)	75 (22.87)	75 (15.59)	
Behavioural characteristics				
Social isolation				0.207
Mostly integrated	173 (21.38)	68 (20.73)	105 (21.83)	
Poorly integrated	358 (44.25)	157 (47.87)	201 (41.79)	
Mostly isolated	278 (34.36)	103 (31.40)	175 (36.38)	
Weekly physical activity				0.008
Inactive or less active	381 (47.10)	154 (46.95)	227 (47.19)	
Moderately active	245 (30.28)	84 (25.61)	161 (33.47)	
Highly active	183 (22.62)	90 (27.44)	93 (19.33)	
Smoking status				<0.001
Never smokers	404 (49.94)	122 (37.20)	282 (58.63)	
Former smokers	324 (40.05)	175 (53.35)	149 (30.98)	
Current smokers	81 (10.01)	31 (9.45)	50 (10.40)	
Health-related characteristics				
Waist circumference (cm)	93.37 (12.80)	99.57 (10.79)	89.14 (12.34)	0.009
Polypharmacy (>5 drugs/day)	201 (24.85)	97 (29.57)	104 (21.62)	0.010
Depressive symptoms	161 (19.90)	28 (8.54)	133 (27.65)	<0.001
MMSE (mean, SD)	28.16 (1.95)	28.34 (1.59)	28.03 (2.16)	0.028
Dietary information				
Daily energy intake (Kcal)	1987.92 (492.26)	2155.15 (509.93)	1873.88 (445.67)	<0.001
Total energy % (mean, SD) from:				
NOVA1	37.31 (8.20)	35.49 (8.02)	38.56 (8.09)	<0.001
NOVA2	14.06 (4.75)	13.05 (4.66)	14.75 (4.69)	<0.001
NOVA3	27.79 (9.01)	30.24 (9.33)	26.11 (8.39)	<0.001
NOVA4	20.84 (8.96)	21.22 (8.80)	20.59 (9.07)	0.327

Abbreviation: MMSE, Mini-mental State Examination. Numbers are frequencies and percentages unless specified.

**Table 2:** Linear regression coefficients (B), standard errors (SEs), and *p*-values for associations between NOVA group (alr) and cognitive domains tests (z-scores) in all participants (n = 809) and by gender.

	NOVA1 vs. NOVA4		NOVA2–3 vs. NOVA4		NOVA4 vs. NOVA1	
Cognitive Tests Domains	Coefficient	SE	Coefficient	SE	Coefficient	SE
**Memory**						
Free and Cued Selective Reminding Test (FCSRT) immediate free recall #	0.162 **	0.077	−0.106	0.067	−0.056	0.046
Men	0.103	0.083	−0.092	0.082	−0.011	0.066
Women	0.214 **	0.080	−0.132	0.082	−0.081	0.054
Free and Cued Selective Reminding Test (FCSRT) immediate total recall #	0.201 **	0.082	−0.081	0.072	−0.120 **	0.049
Men	0.191 **	0.099	−0.093	0.067	−0.097	0.064
Women	0.190 *	0.100	−0.058	0.083	−0.133 **	0.050
Free and Cued Selective Reminding Test (FCSRT) delayed free recall #	0.176 **	0.078	−0.066	0.068	−0.110 **	0.047
Men	0.126	0.086	−0.051	0.083	−0.075	0.067
Women	0.222 **	0.082	−0.093	0.084	−0.128 **	0.052
Free and Cued Selective Reminding Test (FCSRT) delayed total recall	0.146 *	0.082	−0.060	0.071	−0.086 *	0.051
Men	0.129	0.090	−0.113	0.081	−0.016	0.086
Women	0.111	0.087	0.018	0.090	−0.129	0.118
Logical Memory Test #	−0.006	0.073	0.043	0.063	−0.037	0.044
Men	−0.090	0.080	0.020	0.076	0.069	0.061
Women	0.059	0.075	0.040	0.074	−0.099 **	0.051
Rey–Osterrieth Complex Figure Test (ROCF)—delayed recall #	0.126	0.077	−0.091	0.067	−0.035	0.046
Men	0.208 **	0.083	−0.196 **	0.091	−0.012	0.062
Women	0.036	0.072	0.020	0.076	−0.056	0.053
**Executive function**						
Frontal Assessment Battery (FAB)	−0.092	0.074	0.096	0.065	−0.005	0.045
Men	−0.118	0.087	0.108	0.086	0.009	0.057
Women	−0.063	0.082	0.076	0.086	−0.012	0.055
Trail Making Test, part B-A (sensitivity)	−0.013	0.075	−0.002	0.065	0.015	0.045
Men	−0.016	0.072	0.077	0.068	−0.061	0.053
Women	0.010	0.066	−0.076	0.076	0.065	0.050
Phonemic verbal fluency #	0.070	0.072	−0.033	0.063	−0.037	0.043
Men	0.017	0.081	−0.044	0.080	0.027	0.056
Women	0.100	0.071	−0.025	0.075	−0.074	0.052
Semantic verbal fluency	0.013	0.082	−0.009	0.071	−0.003	0.049
Men	0.042	0.048	0.002	0.073	−0.044	0.066
Women	0.003	0.041	−0.023	0.066	0.020	0.059
**Language**						
Picture Naming Test #	0.178 **	0.076	−0.114 *	0.066	−0.064	0.046
Men	0.212 **	0.081	−0.124 **	0.061	−0.088 *	0.051
Women	0.157 **	0.076	−0.106	0.085	−0.052	0.069
**Visuospatial abilities**						
Rey–Osterrieth Complex Figure Test (ROCF)—copy	0.119	0.082	−0.137 *	0.072	0.018	0.049
Men	0.170	0.163	−0.251	0.273	0.081	0.122
Women	0.039	0.059	−0.013	0.061	−0.027	0.038
**Global cognitive score**						
(Memory + executive + language + visuospatial) #	0.158 **	0.073	−0.079 *	0.059	−0.079 *	0.043
Men	0.145 *	0.078	−0.127 *	0.071	−0.018	0.054
Women	0.152 **	0.072	−0.035	0.073	−0.117 **	0.052

Legend: SE: Standard error; alr: additive log-ratio; # Gender statistically significant interaction. *p* < 0.10 (*). *p* < 0.05 (**). The models include terms for age, gender, education, practising sports, smoking, depressive symptoms, daily number of drugs, total energy intake, waist circumference, and social isolation.

## Data Availability

The data presented in this study are available on request from the corresponding author. The data are not publicly available due to privacy and ethical restrictions to protect patient confidentiality.

## Supplementary Materials

The following supporting information can be downloaded at https://www.mdpi.com/article/10.3390/nu18132074/s1, Table S1: Food items considered in the four NOVA groups according to the NOVA-classification in the NutBrain cohort; Table S2: Linear regression coefficients (B), standard errors (SEs), and *p*-values for associations between NOVA groups (alr) and global cognitive function (MMSE); Table S3: Linear regression coefficients (B), standard errors (SEs), and *p*-values for associations between NOVA groups (alr) and global cognitive function (MMSE) in participants with MMSE greater than 22 (n = 797).

## Abbreviations

The following abbreviations are used in this manuscript:

UPFs	Ultra-processed foods
CoDa	Compositional data analysis
NutBrain	Nutrition, Gut Microbiota, and Brain Aging
MMSE	Mini-Mental State Examination
FCSRT	Free and Cued Selective Reminding Test
ROCF	Rey–Osterrieth Complex Figure Test
FAB	Frontal Assessment Battery
TMT	Trail Making Test
SD	Standard deviation
SFFQ	Semi-quantitative food frequency questionnaire
BEST	Bollate Eye Study
alr	Additive log-ratios
CES-D	Centre for Epidemiological Studies Depression Scale
IPAQ	International Physical Activity Questionnaire
CRIq	Cognitive Reserve Index Questionnaire
SE	Standard error
