# Supplementary material for: Patterns of Ultra-Processed Food Consumption and Cognitive Performance in Older Adults: A Population-Based Cross-Sectional Analysis from Northern Italy
Source: Nutrients. 2026 Jun 24;18(13):2074. doi: 10.3390/nu18132074 (PMC13363482; doi:10.3390/nu18132074)
Supplement: Supplementary file 1 [file nutrients-18-02074-s001.zip › nutrients-4365423-supplementary.pdf]

Supplementary Table S1. Food items considered in the four NOVA groups according to the NOVA-classification in the NutBrain cohort.

Dry pasta, rice, potatoes, egg, sliced beef, veal, horse, pork chop, white meat, lamb, kid, offal, legumes, macro fish, fat fish, blue fish, crustaceans/shellfish, whole milk, skim milk skim milk, low-fat and whole yoghurt, raw leaf vegetables, tomatoes, fennel, green beans, asparagus, cucumbers, courgettes, beetroot, carrots, peppers, artichokes, cauliflower/broccoli/ cabbage, onions, mushrooms, aubergines, spinach, minestrone, apricot, whole or squeezed orange, banana, cherry, watermelon/melon, strawberry/raspberry/blueberry, tangerine/mandarin orange, pear, apple, peach, kiwi, plum, grape, dried fruit, tomato sauce, water, coffee, tea/tea.	**NOVA 1**
Butter, margarine, seed oil, olive oil, sugar, honey, salt	**NOVA 2**
Bread, pizza, rusks, egg pasta, spaghetti or rice in broth, pasta and beans/lentils/peas, potato gnocchi, lasagne/cannelloni, polenta, tuna in oil, parmesan, grana, pecorino, ricotta, cottage cheese, mozzarella, provola, scamorza, other cheeses, meat sauce, beer, red wine, white wine	**NOVA 3**
Crackers, ordinary biscuits, breadsticks, ravioli, tortellini, raw ham, speck, fresh sausage, cooked ham, bresaola, other sliced meats, crescenza cheese, spreadable cheese, cream ice cream, fruit ice cream, brioche, pastries, cake, candy, chocolate, bagged crisps, lard, sweet drinks, fruit juices, bitters or liqueurs, sweeteners, jam	**NOVA 4**

Supplementary Table S2. Linear regression coefficients (B), standard errors (SE), and p-values for associations between NOVA group (alr) and global cognitive function (MMSE)

	All (n=809)			Men (n=328, 40.5%)			Women (n=481, 59.5%)		
**NOVA alr**	**Coefficient**	**SE**	**p-**	**Coefficient**	**SE**	**p-value**	**Coefficient**	**SE**	**p-**
NOVA1 vs others	0.36*	0.15	0.014	0.34*	0.15	0.026	0.40*	0.15	0.008
NOVA2-3 vs others	-0.10	0.12	0.417	-0.04	0.13	0.786	-0.16	0.16	0.319
NOVA 4 vs others°	-0.26*	0.09	0.003	-0.31*	0.11	0.004	-0.23*	0.11	0.030

SE, Standard error; MMSE, Mini-Mental State Examination; alr, additive log-ratio. The models also include terms for age, gender, education, practising sports, smoking, depressive symptoms, daily number of drugs, total energy intake, waist circumference, and social isolation.*p-value < 0.05. °Obtained in a second model that included the two alr with NOVA1 as the denominator.

Supplementary Table S3. Linear regression coefficients (B), standard errors (SE), and p-values for associations between NOVA group (alr) and global cognitive function (MMSE) in participants with MMSE greater than 22 (n = 797)

	All (n=797)			Men (n=328, 41.2%)			Women (n=469, 58.9%)		
**NOVA alr**	**Coefficient**	**SE**	**p-**	**Coefficient**	**SE**	**p-value**	**Coefficient**	**SE**	**p-**
NOVA1 vs others	0.22	0.09	0.067	0 .19	0.13	0.146	0.31*	0.13	0.016
NOVA2-3 vs others	-0.06	0.10	0.556	-0.05	0.12	0.669	-0.20	0.14	0.147
NOVA 4 vs others°	-0.16*	0.07	0.031	-0.24*	0.10	0.016	-0.11	0.09	0.211

SE, Standard error; MMSE, Mini-Mental State Examination; alr, additive log-ratio. The models also include terms for age, gender, education, practising sports, smoking, depressive symptoms, daily number of drugs, total energy intake, waist circumference, and social isolation. .*p-value < 0.05. °Obtained in a second model that included the two alr with NOVA1 as the denominator.
